# Long-term Outcomes of Lupus Nephritis in Comparison to Other CKD Etiologies

**DOI:** 10.1016/j.ekir.2024.10.021

**Published:** 2024-10-28

**Authors:** Charikleia Chrysostomou, Francesca Faustini, Iva Gunnarsson, Mårten Segelmark, Juan-Jesús Carrero, Peter Barany, Anne-Laure Faucon, Marie Evans

**Affiliations:** 1 Division of Rheumatology, Department of Medicine-Solna, Karolinska Institutet, Karolinska University Hospital, Stockholm, Sweden; 2 Department of Clinical Sciences, Lund University, Lund, Sweden; 3 Department of Nephrology, Skåne University Hospital, Malmö-Lund, Sweden; 4 Department of Medical Epidemiology and Biostatistics, Karolinska Institutet, Stockholm, Sweden; 5 Division of Renal Medicine, Department of Clinical Science, Intervention and Technology, Karolinska Institutet, Stockholm, Sweden; 6 INSERM UMR 1018, Department of Clinical Epidemiology, Centre for Epidemiology and Population Health, Paris-Saclay University, France; 7 Department of Nephrology, Karolinska University Hospital, Stockholm, Sweden

**Keywords:** cardiovascular event, chronic kidney disease, kidney replacement therapy, lupus nephritis, mortality, primary glomerular disease

## Abstract

**Introduction:**

Little is known regarding the comparison of cardiovascular and kidney outcomes between lupus nephritis (LN) and other etiologies of chronic kidney disease (CKD).

**Methods:**

Using data from the Swedish Renal Registry (2006–2021), we compared long-term outcomes between patients with LN-CKD and patients with CKD due to primary glomerular diseases (PGD) and other CKD causes (Other-CKD, mainly diabetes and nephroangiosclerosis). Adjusted hazard ratios (HRs) of mortality, major adverse cardiovascular events (MACE) and kidney replacement therapy (KRT) were estimated using Cox proportional hazard models.

**Results:**

At baseline, LN (*n* = 317, 61 years, 76% women) and PGD (*n* = 2296, 57 years, 30% women) had better kidney function and lower prevalence of cardiovascular disease than the Other-CKD (*n* = 34,778, 75 years, 36% women). The median follow-up was 6.2 (3.3–9.8) years. The absolute risks of death and MACE in LN-CKD were intermediate between those of Other-CKD and PGD. The 5-year absolute KRT risk of LN-CKD was similar to Other-CKD’s risk (22%) and lower than in PGD (37%). In multivariable analysis, as compared to PGD, the rates of death and MACE in LN-CKD were higher (HR: 1.63 [95% confidence interval: 1.32–2.02] and 1.65 [1.31–2.08]), whereas the rate of KRT tended to be lower (0.81 [0.64–1.02]). In contrast, the rate of adverse events was not different between LN-CKD and Other-CKD.

**Conclusion:**

Although patients with LN-CKD had a lower risk of KRT than PGD-CKD, they exhibited higher risk of death and MACE, reaching the risk magnitude of patients with high cardiovascular burden (Other-CKD). Our findings may inform decisions about prevention of cardiovascular events in patients with moderate and advanced LN-CKD.

## Graphical abstract

**Figure:**
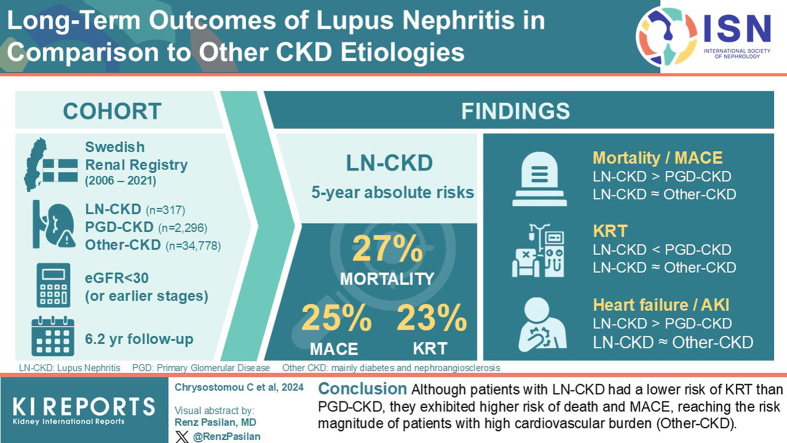

Systemic lupus erythematosus (SLE) is a chronic, multiorgan, autoimmune disease associated with substantial morbidity and mortality.^1^ LN, a severe manifestation of SLE caused by nephron inflammation, often leads to CKD.^2^ Despite major improvements in the understanding of the pathophysiology, diagnosis, and clinical management of SLE and LN, patients with SLE/LN-CKD still have an elevated risk of developing adverse clinical outcomes, such as kidney failure,^3^ cardiovascular disease,^4^ and death.^5^, ^6^, ^7^

Several large-scale studies on kidney replacement therapy (KRT)- populations have shown that SLE/LN is associated with up to 2-fold higher risk of cardiovascular events and mortality when compared to other diagnoses of kidney failure;^8^, ^9^, ^10^, ^11^ however, data regarding the comparison of non-dialysis patients with SLE/LN-CKD to other CKD etiologies are limited. It is well-known that CKD *per se* is a major risk factor for kidney failure, cardiovascular disease, and premature mortality.^12^^,^^13^ However, whether and how the diagnosis of SLE/LN might further affect the prognosis of patients with moderate and advanced CKD has not been adequately investigated so far. Few previous investigations suggest that patients with SLE/LN-CKD might be at higher risk of KRT, ischemic events, and death in contrast to patients with other CKD causes. However, those studies were based on small sample sizes^14^ with limited follow-up,^15^ did not evaluate SLE/LN separately from other causes of glomerulonephritis,^16^ or yielded conflicting results.^14^, ^15^, ^16^, ^17^, ^18^ Finally, in some studies, the comparator group might have been inadequate: comparing LN-CKD to SLE without kidney disease may not allow separating the risk attributed to LN from that due to the overall burden of CKD.^19^

Recognizing CKD etiologies with a particularly elevated risk of adverse events is crucial.^20^^,^^21^ Therefore, we decided to compare the risks of cardiovascular and renal outcomes between patients with SLE/LN-CKD and those with other CKD etiologies, using data from a nationwide registry of patients referred to nephrologist care.

## Methods

### Data Source and Study Population

This study was performed with data from patients with CKD included in the Swedish Renal Registry (SRR), a nationwide registry which has enrolled more than 51,000 patients with CKD^1^, ^2^, ^3^, ^4^, ^5^^,^^22^ (from various etiologies) attending routine nephrological care. Registration into SRR is mandatory when the estimated glomerular filtration rate (eGFR) is <30 ml/min per 1.73 m^2^; however, nephrological centers are also encouraged to register patients earlier in the course of the disease, for example, when eGFR is < 45 to 60 ml/min per 1.73 m^2^, or to enter all their patients with CKD, as long as they fulfill the same selected GFR criteria. Of the nephrology departments in Sweden, 97% participate and it has been estimated that 75% to 90% of patients with CKD stage 4 to 5 attending nephrological care are registered in the SRR.^23^ After inclusion, a minimum of 1 outpatient nephrology visit is registered every year, until death, start of KRT, or emigration from the country. Via each citizen’s unique personal identification number, the Swedish Renal Registry CKD is linked to other government-run registries, including the National Patient Registry,^24^ which provides information on all out-patient specialist consultations and hospitalizations occurring in Swedish health care system since 1997; the Prescribed Drug Registry,^25^ which records information on prescribed drugs dispensed at any Swedish pharmacy, and the National Death Registry,^24^ which provides information on date and causes of death. At enrollment, patients receive oral and written information. The Regional Ethical Review Boards and the Swedish National Board of Welfare approved the study (project numbers 2018/1591-31/2 and 2022-04594).

In this study, we included, between January 1, 2006, and December 31, 2021, patients aged > 15 years, with CKD and a previous diagnosis of SLE and/or LN (SLE/LN-CKD), PGD-CKD, and CKD attributed to a non-inflammatory etiology (Other-CKD). Patients with a previous history of kidney transplantation, on maintenance dialysis, or without any eGFR evaluation were excluded (Supplementary Figures S1 and S2). The index date was defined as the cohort entry date and constituted the start of follow-up.

### Exposure and Comparison Groups

At inclusion into the SRR, CKD etiology is assigned by a clinical nephrologist according to the European Renal Association (ERA) coding system.^26^ Since 2012, the coding system also includes information on whether the diagnosis is histologically proven or not. In our study and in order to minimize misclassification error, we included patients from Swedish Renal Registry CKD with a diagnosis of LN according to the ERA classification and/or a clinical diagnosis of SLE/LN defined by the ICD-10 codes M32.1, M32.9, or M32.8 from the interlinked Patient Registry, according to a previous validated identification of SLE in Sweden.^27^ All patients with an ICD-10 code of SLE/LN inconsistent with SLE/LN-CKD diagnosis according to ERA-classification system were independently adjudicated by 2 nephrologists (CC and A-LF) and classified as SLE/LN-CKD only upon agreement.

In order to gain in generalizability and to avoid excluding or misclassifying CKD attributed to more than 1 etiology (e.g., diabetic kidney disease can also include glomerular hyperfiltration and nephroangiosclerotic lesions^28^), we included, in the first comparison group, patients with CKD from various etiologies (Other-CKD) that did not occur in the setting of systemic, autoimmune, inflammatory, infectious disease, solid/hematologic malignancies, or genetic disease (Supplementary Table S1). The Other-CKD group, thus, reflected a broad patient population from the routine clinical practice, including the most common etiologies of CKD, mainly diabetic kidney disease and nephroangiosclerosis, and was considered a group of high cardiovascular risk. The second comparison group consisted of 3 of the most frequent causes of PGD-CKD: primary IgA nephropathy, primary focal segmental glomerulosclerosis and primary membranous nephropathy. Minimal change disease was excluded due to important prognostic differences. The PGD-CKD group was expected to be similar to the SLE/LN-CKD population in terms of baseline age, kidney function, comorbidities; and often characterized by a renal inflammatory state and previous exposure to immunosuppressive treatment.

### Covariates

Covariates were extracted from the National registries and the Swedish Renal Registry and were derived at index date. Detailed definitions of each covariate are presented in Supplementary Table S2. They included age, sex, baseline comorbidities (hypertension; diabetes mellitus; dyslipidemia; ischemic heart disease; peripheral arterial disease; cerebrovascular disease; heart failure; atrial fibrillation; other coagulation disorders-including antiphospholipid syndrome, acute kidney injury [AKI], osteoporosis, and depression or anxiety), clinical data (systolic and diastolic blood pressure and body mass index), laboratory measurements (eGFR estimated by using the 2009 CKD-Epidemiology Collaboration equation and urine albumin-to-creatinine ratio, both expressed according to the Kidney Disease Improving Global Outcomes [KDIGO] categories; hemoglobin; and serum albumin), ongoing medications dispensed within the 6 months prior to index date (renin-angiotensin-system-inhibitors, corticosteroids, oral immunosuppressive drugs, aspirin, anticoagulants, and lipid lowering drugs) and health care utilization (number of hospitalizations and all-cause hospitalizations) within 12 months prior to index date. The diagnosis vintage of patients with SLE/LN-CKD and PGD-CKD was estimated as the time between the first diagnosis (according to administration claims) and the date of the first registered visit in SRR.

### Outcomes

The primary outcomes included KRT, defined as dialysis or preemptive kidney transplantation; MACE, a composite of cardiovascular death, myocardial infarction, or stroke); and all-cause mortality. The secondary outcomes included the single components of MACE, heart failure, and AKI as in-hospital diagnosis (Supplementary Table S2). Patients were followed-up with until the occurrence of event, death, or the end of follow-up on December 31, 2021.

### Statistical Analyses

Continuous variables were reported as median with interquartile range. Categorical variables are reported as frequencies and percentages.

Cumulative incidence curves of each outcome were estimated using the Aalen-Johansen approach, with death treated as a competing risk. The Aalen Johansson approach was preferred to the Kaplan-Meier method because it is more appropriate when competing risks are present.^29^, ^30^, ^31^

Crude and adjusted HRs of each outcome associated with SLE/LN-CKD, as compared to PGD-CKD or to Other-CKD, were estimated using Cox proportional hazards models. The main analysis was adjusted for several confounders, selected *a priori* on the basis of current knowledge, the literature on this topic,^8^^,^^10^^,^^16^^,^^18^^,^^32^, ^33^, ^34^, ^35^ and by consensus among study authors, including: age, sex, hypertension, diabetes, dyslipidemia, history of ischemic heart disease, cerebrovascular and peripheral artery disease, heart failure, arrhythmia, baseline systolic and diastolic blood pressure, body mass index, baseline serum albumin, eGFR, baseline medication such as renin-angiotensin system inhibitors, aspirin, vitamin K antagonists, direct oral anticoagulants, health care utilization during the last 12 months before inclusion (all-cause hospitalizations and number of hospitalizations), and calendar year. Considering that albuminuria, a feature of kidney disease and a strong predictor for adverse outcomes,^23^ is on the causal pathway between etiology of kidney disease and adverse outcomes (i.e., urine albumin-to-creatinine ratio is considered as a mediator rather than a confounding factor), it was not included in the main analysis. Finally, we tested the interaction between the exposure (SLE/LN-CKD) and sex in our multivariable Cox models. For each model, the functional relationship between continuous covariates and outcomes were analyzed using Martingale residuals.^36^ The proportional hazards assumption was checked using log(–log[S]) plots and Schoenfeld residuals against time.

Missing data (systolic and diastolic blood pressure and serum albumin) were mostly around 5% except for urine albumin-to-creatinine ratio, which was missing in 29% of patients. Missing data were assumed to be missing at random due to lack of reporting into the registry, because they are part of the routine monitoring practice in CKD. We thus performed multiple imputations using the chained equation algorithm (50 datasets generated with 20 iterations). Cumulative hazard rate (by using the Nelson-Aalen estimator^31^) and all variables from the fully adjusted Cox model were considered for the imputation procedure.^37^ Results from the 50 Cox models were then pooled using the Rubin’s rule.

### Sensitivity Analyses

In addition, as sensitivity analyses, models were further adjusted for albuminuria. We also re-ran the models, restricting the SLE/LN-CKD group to patients with a LN diagnosis according to the ERA-classification system (*n* = 218/317).

The STROBE statement was followed for reporting observational studies.^38^ All statistical analyses were conducted using R 4.0.2 software^39^ (R packages: "dplyr", "tidyr" "ggplot2", "mice", "biostat3", "pammtools", "survival", “cmprsk”, “riskRegression”, “adjustedCurves").

## Results

### Baseline Characteristics

We identified 317 patients with SLE/LN-CKD (median age of 61 years, 76% women, median eGFR of 30 ml/min per 1.73 m^2^); and 2296 patients with PGD-CKD (57 years, 30% women, eGFR 29 ml/min per 1.73 m^2^), including 1523 with IgA nephropathy, 394 with focal segmental glomerulosclerosis, and 379 with membranous nephropathy. The Other-CKD group included 34,778 patients (75 years, 36% women, eGFR 25 ml/min per 1.73 m^2^). The baseline characteristics of the study population are displayed in Table 1. As compared to Other-CKD, patients with SLE/LN-CKD and PGD-CKD were younger, had higher eGFR and a lower prevalence of diabetes and cardiovascular disease; whereas they had at baseline a higher exposure to immunosuppressive therapy and a higher level of albuminuria (Table 1, Supplementary Table S7). Diagnosis vintage before inclusion in SRR was longer in patients with SLE/LN-CKD (7.9 [3.7–11.3] years) than in PGD-CKD (4.5 [1.0–8.6] years).

**Table 1:** Baseline characteristics of the study population

Characteristics	NA, %	Overall	Primary glomerular diseases	SLE/LN-CKD	Other-CKD
		*N* = 37,391	*n* = 2296	*n* = 317	*n* = 34,778
Demographics and clinical data					
Age, yrs (Q1–Q3)	0	74.0 (65–81)	57.0 (44–69)	61.0 (46–73)	75.0 (67–81)
Sex, women	0	13,315 (35.6)	687 (29.9)	240 (75.7)	12,388 (35.6)
Body mass index, kg/m^2^ (Q1–Q3)	39.3	27.5 (24.3–31.6)	27.1 (24.1–31.2)	25.2 (22.4–29.4)	27.6 (24.3–31.6)
Systolic BP, mm Hg (Q1–Q3)	5.6	140 (125–150)	132 (120–145)	130 (120–144)	140 (125–151)
Diastolic BP, mm Hg (Q1–Q3)	5.6	77 (70–83)	80 (72–88)	80 (70–85)	76 (70–82)
Comorbidities in medical history					
Hypertension	0	35,801 (95.7)	2164 (94.3)	295 (93.1)	33,342 (95.9)
Diabetes mellitus	0	17,241 (46.1)	383 (16.7)	40 (12.6)	16,818 (48.4)
Dyslipidemia	0	22,450 (60)	1188 (51.7)	110 (34.7)	21,152 (60.8)
Myocardial infarction	0	8244 (22)	140 (6.1)	41 (12.9)	8063 (23.2)
Ischemic heart disease	0	11,434 (30.6)	215 (9.4)	956 (17.7)	11,163 (32.1)
Peripheral vascular disease	0	4630 (12.4)	58 (2.5)	30 (9.5)	4542 (13.1)
Cerebrovascular disease	0	6487 (17.3)	173 (7.5)	47 (14.8)	6267 (18)
Heart failure	0	10,795 (28.9)	177 (7.7)	50 (15.8)	10,568 (30.4)
Atrial fibrillation / flutter	0	8548 (22.9)	211 (9.2)	41 (12.9)	8296 (23.9)
Acute kidney injury	0	6200 (16.6)	262 (11.4)	41 (12.9)	5897 (17)
Other coag. disorders, including APS	0	830 (2.2)	33 (1.4)	46 (14.5)	751 (2.2)
Depression / anxiety	0	3111 (8.3)	201 (8.8)	33 (10.4)	2877 (8.3)
Osteoporosis	0	1322 (3.5)	49 (2.1)	39 (12.3)	1234 (3.5)
Biological data					
Hemoglobin, g/dl (Q1–Q3)	2.6	12.2 (11.1–13.3)	12.6 (11.5–13.9)	12.1 (11.0–13.2)	12.1 (11–13.3)
Albumin, g/l (Q1–Q3)	5.6	37 (34–40)	36 (33–39)	36 (32–39)	37 (34–40)
eGFR, ml/min per 1.73 m^2^ (Q1–Q3)	0.1	25 (18.1–32.1)	28.9 (20.6–43.8)	30 (22.2–44.8)	24.7 (18–31.6)
eGFR, ml/min per 1.73 m^2^, categories	0.1				
>60		1249 (3.3)	325 (14.2)	54 (17)	870 (2.5)
45–59		1748 (4.7)	228 (9.9)	25 (7.9)	1495 (4.3)
30–44		8619 (23.1)	507 (22.1)	79 (24.9)	8033 (23.1)
15–29		19,889 (53.2)	939 (41)	128 (40.4)	18,822 (54.1)
< 15		5866 (15.7)	293 (12.8)	31 (9.8)	5542 (15.9)
ACR, mg/mmol (Q1–Q3)	28.9	24.9 (4.5–119)	90.9 (26–216.1)	43.6 (6.2–123)	21.6 (4.1–109)
ACR categories	28.9				
A1		5006 (18.8)	118 (6.3)	36 (15.4)	4852 (19.8)
A2		9068 (34.1)	386 (20.6)	70 (29.9)	8612 (35.2)
A3		12498 (47)	1374 (73.2)	128 (54.7)	10996 (45)
Medications within 6 mo prior to index date					
RASi	0	24,546 (65.6)	1875 (81.7)	226 (71.3)	22,445 (64.5)
Corticosteroids	0	5448 (14.6)	492 (21.4)	194 (61.2)	4762 (13.7)
MMF	0	387 (1)	55 (2.4)	76 (24)	256 (0.7)
CNI	0	491 (1.3)	81 (3.5)	14 (4.4)	396 (1.1)
Azathioprine	0	254 (0.7)	23 (1)	49 (15.5)	182 (0.5)
Vitamin K antagonists / heparin	0	4826 (12.9)	179 (7.8)	53 (16.7)	4594 (13.2)
Direct oral anticoagulants	0	2003 (5.4)	54 (2.4)	15 (4.7)	1934 (5.6)
Aspirin	0	15,061 (40.3)	368 (16)	79 (24.9)	14,614 (42)
Statin / ezetimib	0	20,394 (54.5)	1095 (47.7)	98 (30.9)	19,201 (55.2)
Health care utilization, within 12 mo prior to index date					
Any hospitalization	0.0	18,112 (48.4)	937 (40.8)	175 (55.2)	17,000 (48.9)
No. of hospitalizations (Q1–Q3)	0.0	0.0 (0.0–9.0)	0.0 (0.0–4.0)	3.0 (0.0–10.0)	0.0 (0.0–9.0)

ACR, albumin-to-creatinine ratio (A1: <3, A2:3–30, A3: >30 mg/mmol); AKI, acute kidney injury; APS, antiphospholipid syndrome; BP, blood pressure; CNI, calcineurin-inhibitor; coag., coagulation; CVD, cardiovascular disease; eGFR, estimated glomerular filtration rate; MMF, mycophenolate mofetil; RASi: renin-angiotensin-system-inhibitor.

### All-cause Mortality, Major Cardiovascular Events, and KRT

**Figure 1:**
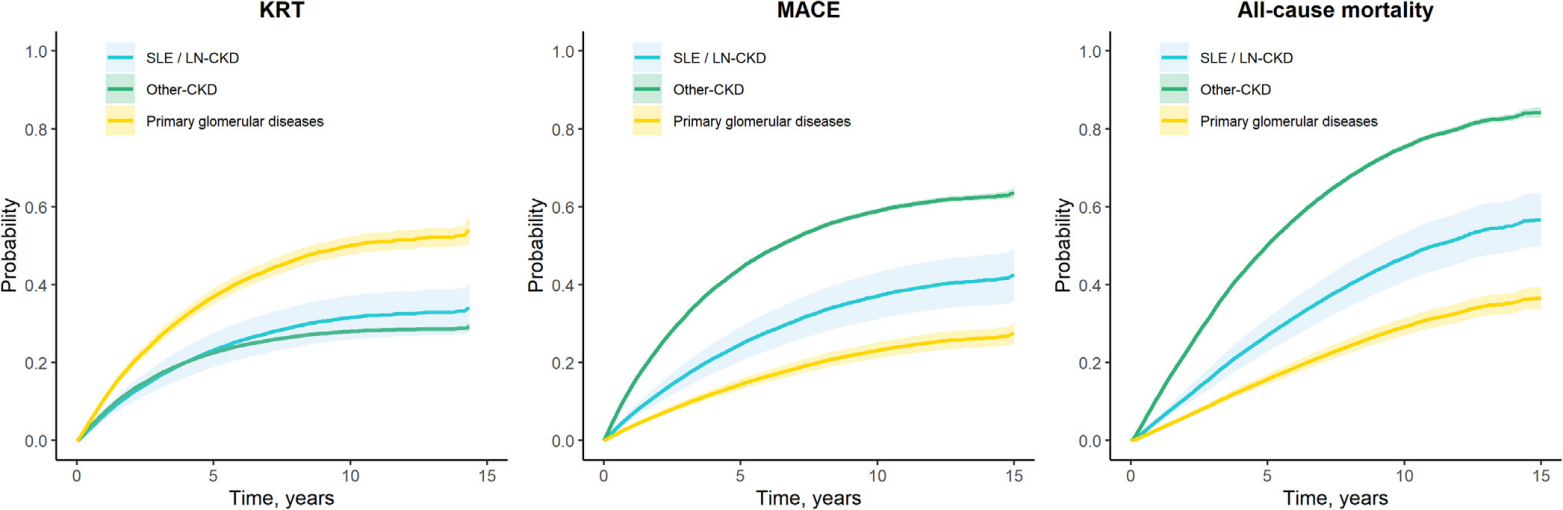
Cumulative incidence curves of adverse clinical outcomes. Crude cumulative incidence curves were estimated using the Aalen-Johansen estimator, with death treated as a competing risk. CKD, chronic kidney disease; KRT, kidney replacement therapy; MACE, major adverse cardiovascular events; SLE/LN, systemic lupus erythematosus / lupus nephritis.

Over a median follow-up of 6.2 (3.3–9.8) years, 19,029 (51%) deaths, 15,768 (42%) MACE, and 8390 KRTs (22%) occurred. The unadjusted 5-year absolute risks of death and MACE of SLE/LN-CKD (27% and 25%, respectively) were intermediate, between those of Other-CKD (50% and 44%) and those of PGD-CKD (16% and 14%); whereas the 5-year risk for KRT was lower in SLE/LN-CKD (23%) than in PGD-CKD (37%), and similar to the KRT-risk of the Other-CKD (22%) (Figure 1 and Supplementary Table S3). The 10-year absolute KRT-risk was 32% for SLE/LN-CKD, 50% for PGD-CKD, and 28% for Other-CKD (Figure 1 and Supplementary Table S3).

**Figure 2:**
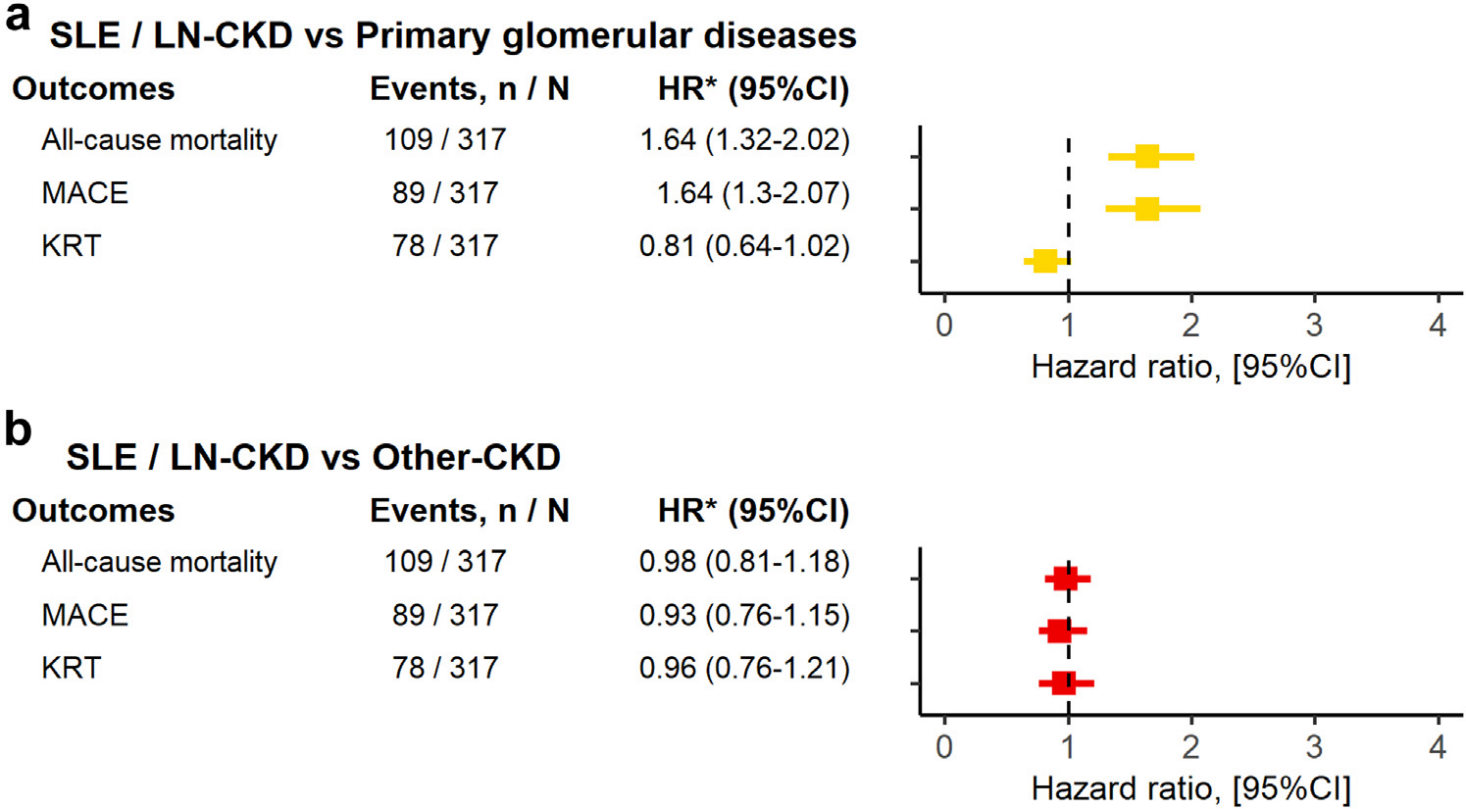
Adjusted hazard ratios of primary outcomes associated with SLE/LN-CKD versus primary glomerular diseases and Other-CKD. Cox models were adjusted for age, sex, hypertension, diabetes, dyslipidemia, history of cardiovascular disease (ischemic heart disease, cerebrovascular disease, peripheral artery disease, heart failure, and arrhythmia), acute kidney injury, systolic and diastolic blood pressure, body mass index, serum albumin, eGFR, renin-angiotensin system inhibitors, aspirin, vitamin K antagonists or heparin, direct oral anticoagulants, health care utilization within 12 months prior to index date (all-cause hospitalizations and number of hospitalizations), and calendar year. CI, confidence interval; CKD, chronic kidney disease; HR, hazard ratio; KRT, kidney replacement therapy; MACE, major adverse cardiovascular events; SLE/LN: systemic lupus erythematosus / lupus nephritis.

In multivariable analyses, as compared to PGD-CKD, SLE/LN-CKD was associated with a higher rate of death (HR: 1.64 [95% confidence interval: 1.32–2.02]) and MACE (HR: 1.64 [1.3–2.07]). However, the rates of death and MACE were similar between SLE/LN-CKD and Other-CKD (HR: 0.98 [0.81–1.18] and HR: 0.93 [0.76–1.15], respectively) (Figure 2 and Supplementary Table S4). The risk estimate of KRT indicated a lower risk in patients with SLE/LN-CKD than in PGD-CKD (HR: 0.81 [0.64–1.02], 95% confidence interval slightly overlapped). The SLE/LN-CKD and the Other-CKD group exhibited similar adjusted risk-magnitude for KRT (HR: 0.96 [0.76–1.21]) (Figure 2 and Supplementary Table S4).

In the sex- interaction analysis, we observed that the relationships between SLE/LN-CKD and the risks of all-cause mortality and MACE were similar in men and women, when compared to PGD and Other-CKD (*P*-value for interaction not significant). This was not the case for the risk of KRT (*P*-value: 0.03 for both SLE/LN vs. PGD and SLE/LN vs. Other-CKD). In women, the risk of KRT was similar between SLE/LN-CKD and the control groups. In contrast, in men, the risk of KRT in SLE/LN-CKD was lower than in PGD-CKD and in Other-CKD (Supplementary Table S8).

### Secondary Outcomes and Sensitivity Analyses

**Figure 3:**
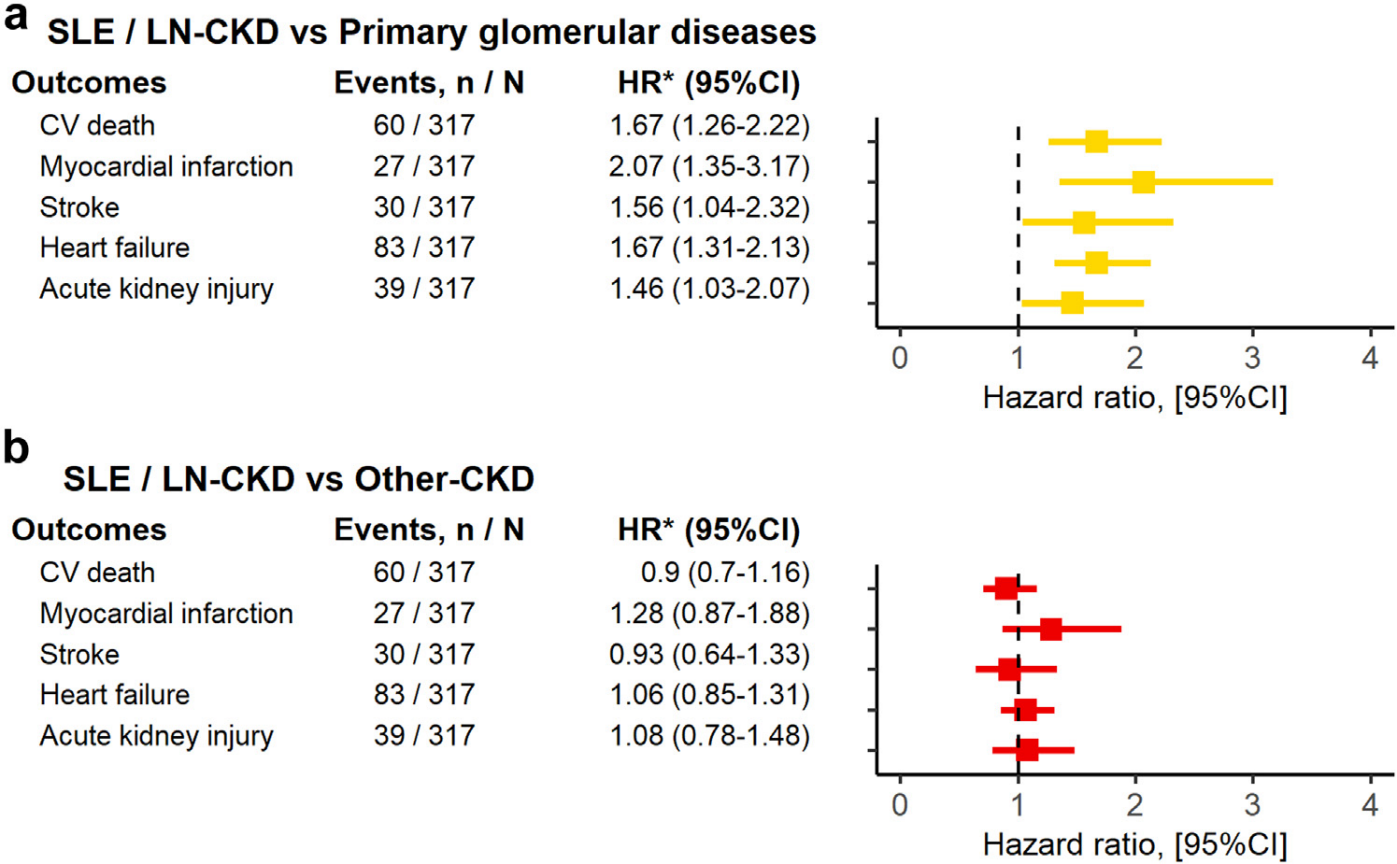
Adjusted hazard ratios of secondary outcomes associated with SLE/LN-CKD versus primary glomerular diseases and Other-CKD. Cox models were adjusted for age, sex, hypertension, diabetes, dyslipidemia, history of cardiovascular disease (ischemic heart disease, cerebrovascular disease, peripheral artery disease, heart failure, and arrhythmia), acute kidney injury, systolic and diastolic blood pressure, body mass index, serum albumin, eGFR, renin-angiotensin system inhibitors, aspirin, vitamin K antagonists or heparin, direct oral anticoagulants, health care utilization within 12 months prior to index date (all-cause hospitalizations and number of hospitalizations), and calendar year. CI, confidence interval; CKD, chronic kidney disease; CV, cardiovascular; HR, hazard ratio; SLE/LN, systemic lupus erythematosus / lupus nephritis.

The adjusted rate of heart failure was higher in SLE/LN-CKD than in PGD-CKD (HR: 1.67 [1.31–2.13]), whereas it was comparable to the Other-CKD (HR: 1.06 [0.85–1.31]). The adjusted rates of the single components of MACE were consistently higher in SLE/LN-CKD than in PGD-CKD, whereas they were not different between SLE/LN-CKD and Other-CKD. (Figure 3 and Supplementary Table S5).

A higher rate of AKI was observed in SLE/LN-CKD when compared to PGD-CKD (HR: 1.46 [1.03–2.07]). In contrast, there was no statistical difference, regarding the AKI rate, between SLE/LN-CKD and Other-CKD (HR: 1.08 [0.78–1.48]) (Figure 3, Supplementary Table S5).

Similar results were observed, in general, after adjustment for albuminuria (Supplementary Table S6). After restricting patients with SLE/LN-CKD to those with a LN diagnosis according to the ERA-classification system, the results from the sensitivity analyses were generally consistent with the main analysis (Supplementary Table S6).

## Discussion

In this large nationwide study of patients with moderate and advanced CKD, receiving longitudinal nephrological care, SLE/LN-CKD was associated with higher risks of cardiovascular events and mortality when compared to PGD-CKD. Furthermore, the adjusted risks of MACE and death of patients with SLE/LN-CKD were similar to those of patients with CKD with high underlying cardiovascular burden (Other-CKD). Our results also showed that, SLE/LN-CKD had a high 5- and 10-year absolute risk for KRT, (23% and 32%, respectively), although it tended to be lower than that of PGD-CKD. This highlights the need for improved strategies to manage the risks and consequences attributed to SLE/LN-CKD.

Our findings expand those of previous epidemiological studies conducted in patients with SLE/LN on maintenance dialysis^9^ and with kidney transplant,^8^^,^^10^^,^^11^ which observed poorer clinical outcomes in LN, as compared to the other CKD-causes leading to KRT.^8^, ^9^, ^10^^,^^40^ Previous studies comparing LN to SLE without kidney disease^5^^,^^19^^,^^41^ or to the general population^42^^,^^43^ were also generally consistent with our results; although these studies were unable to separate the effect attributed to LN from the risks associated to the overall burden of CKD. By choosing as comparison group patients with various CKD etiologies, we overcame this limitation, allowing us to better understand the impact of SLE/LN *per se* on adverse outcomes. Thus, our findings support that the etiology of CKD itself might add value for risk stratification, over and above eGFR and albuminuria.^33^

There is a small body of literature comparing LN to other CKD etiologies.^14^, ^15^, ^16^, ^17^, ^18^ In a small retrospective observational study conducted on 185 patients with biopsied CKD (82 patients with LN), the risk of all-cause mortality was not significantly different between patients with LN, crescentic glomerulonephritis, and diabetic kidney disease.^14^ A few previous, small-scaled, studies have evaluated outcomes of patients with different types of glomerulonephritis mixed into 1 group (including LN). Hutton *et al.*^15^ showed that the risk of cardiovascular event was similar between glomerulonephritis and the other causes of CKD, whereas other studies demonstrated a lower mortality risk in patients with glomerulonephritis when compared to diabetic kidney disease.^16^, ^17^, ^18^ However, these studies cannot enable disentangling the risks specifically attributed to SLE/LN from the other types of glomerulonephritis.

In our study, we observed that the adjusted risks of MACE, heart failure, and all-cause mortality in SLE/LN-CKD were similar to those of the Other-CKD group and higher than those of the PGD-CKD. Several possible pathophysiological mechanisms might explain these observations. SLE/LN is characterized by a chronic inflammatory state,^44^ which together with a chronic exposure to long-term immunosuppressive medications^45^ and coagulation or vascular disorders in the setting of antiphospholipid syndrome,^46^^,^^47^ lead to endothelial dysfunction and accelerated atherosclerosis.^48^^,^^49^ When compared to PGD-CKD and Other-CKD, a higher proportion of the patients with SLE/LN-CKD in our cohort had coagulation disorders (including antiphospholipid syndrome), which may have mediated some of their higher risk for adverse cardiovascular outcomes. In patients with SLE/LN with advanced CKD, antimalarial therapy remains underprescribed and is often discontinued, probably because of concerns regarding drug accumulation and toxicity^50^ even though the European League Against Rheumatism guidelines strongly recommends antimalarial utilization^46^ to improve the cardiovascular prognosis. We also noted that some nephro-cardio-protective treatments such as renin-angiotensin-system-inhibitors and statins were less often prescribed to the patients with SLE/LN-CKD of our cohort. However, the difference in baseline renin-angiotensin-system-inhibitors and lipid-lowering drug prescriptions does not explain the observed results, because the models were adjusted for these medications. Finally, the high prevalence of anxiety or depression, and nonadherence to treatment,^51^^,^^52^ both in SLE and in CKD in general, further complicate the clinical management of patients with SLE/LN-CKD, and might contribute, at least in part, to their excess risk for adverse clinical outcomes.

Further analysis of our data showed that SLE/LN-CKD exhibited a high 5-and 10-year absolute KRT risk (23% and 32%, respectively), which is in accordance with the literature.^3^^,^^8^^,^^41^^,^^53^ We also observed a higher relative rate of AKI in SLE/LN-CKD when compared to PGD-CKD. The high risk of AKI and KRT observed in SLE/LN-CKD might be explained by an elevated risk of thrombotic microangiopathy,^54^^,^^55^ crescentic injury,^56^ flare or relapse, kidney damage accrual,^57^ and infection complications due to immunosuppressive treatment.^58^ Surprisingly, in the multivariable analyses, even after adjustment for albuminuria, we observed that patients with SLE/LN-CKD tended to have a lower KRT-risk than patients with PGD-CKD. This would suggest that other factors, intrinsic to kidney disease etiology and its clinical management, might explain the remaining difference. It is well-known that patients with autoimmune focal segmental glomerulosclerosis, who have lower eGFR levels, are often those characterized by resistance to immunological treatment and have poor renal prognosis,^59^^,^^60^ and that patients with IgA nephropathy with moderate to advanced CKD have a substantial KRT risk,^23^^,^^60^, ^61^, ^62^ without, up to recently, effective immunosuppressive treatment possibilities.^63^^,^^64^

The strengths of our study include the direct comparison of a large number of well-characterized patients with SLE/LN-CKD to patients with the most common etiologies of CKD reflecting routine clinical practice; the unique setting of a national representative registry with long-term follow-up; the publicly-funded Swedish health care system, which enables avoiding selection bias from differential access to medical care; and the analysis of a large set of clinical outcomes with no loss to follow-up, due to the data acquired from the national registries. Our study has some limitations, including its observational nature, which is prone to residual confounding. Second, we were not able to include patients with SLE/LN not receiving nephrological care, and we cannot rule out the possibility of misclassification of patients who were identified by ICD-codes of SLE/LN. However, most patients with the range of eGFR included in our study (CKD Stages 3b–5) are usually under nephrological care. By combining different sources of coding systems and by adjudicating all diagnoses with discrepancies, we minimized any potential misclassification bias, and the results were consistent when analyses were restricted to patients with LN with an ERA-registry diagnosis. Third, information on SLE/LN, especially on systemic activity, kidney histopathological features, data on antimalaria treatment, and i.v. immunosuppressive therapies were not available in our data sources. These data could, however, be regarded as mediators (indirect effects associated with the CKD-etiology itself), not as confounders, and including them in the models would mean adjusting in the causal pathway. In addition, patients were included at the time of their first registration in the registry, and not necessarily at the time of diagnosis. This probably did not compromise the internal validity of our results, because the registry criteria for inclusion were similar for all comparison groups. The diagnosis vintage observed in patients with SLE/LN was longer than in PGD, which can be explained by the fact that SLE is a systemic disease than may involve nonkidney manifestation,^65^ and LN usually develops within some years from the SLE onset.^66^ Registration of race is not allowed in the Swedish national registries due to the Swedish legislation; however, from our clinical experience, we assumed that the majority of patients were of Caucasian origin. However, although the lack of ethnic diversity might constitute a limitation of our study, it does not compromise the internal validity of our findings. Finally, our study represents high renal-risk patients with mainly moderate and advanced CKD (stage 3b–5), which therefore might preclude generalizability of our findings to higher stage of kidney function.

Our findings have clinical implications. First, they highlight the importance of acknowledging and defining CKD etiologies with particularly high risk of adverse events. In line with previous studies^67^ and highlighted by the KDIGO-guidelines,^68^ the underlying nephropathy constitutes one of the most important independent predictors of outcomes. Second, our findings emphasize the prognostic significance of cardiovascular and renal prevention in SLE/LN-CKD. According to the European League Against Rheumatism recommendations for the management of cardiovascular risk in SLE^46^ and the recent European League Against Rheumatism (EULAR)- and KDIGO- LN treatment guidelines,^69^, ^70^, ^71^ antimalarials, even in later CKD stages, under therapeutic drug monitoring,^46^^,^^72^ and the intensification of cardioprotective and nephroprotective treatments, such as renin-angiotensin-system-inhibitors and sodium-glucose transport protein 2-inhibitors,^63^^,^^73^ novel mineralocorticoid receptor antagonists,^74^ and glucagon-like peptide-1-receptor agonists^75^^,^^76^ could improve the prognosis of SLE/LN-CKD. Furthermore, severe infections in later stages of CKD are associated with a negative cardiovascular and renal impact;^77^ so minimizing the steroid dose in SLE-CKD^71^^,^^78^, ^79^, ^80^ might mitigate both the infection- and the cardiovascular risks. Finally, our results emphasize the need for additional epidemiologic and pharmaco-epidemiologic research in SLE/LN across all stages of CKD.

In conclusion, our study demonstrates that patients with SLE/LN-CKD are at high risk of adverse clinical outcomes, including KRT, cardiovascular events, and all-cause mortality. Periodical screening for cardiovascular risk factors, a greater focus on individualized clinical management toward precision medicine and the implementation of SLE/LN-specialized multidisciplinary teams are needed to improve the prognosis in this population.

## Disclosure

ME reports personal honoraria for lectures by AstraZeneca, Astellas pharma, Vifor Pharma, Fresenius healthcare, and Baxter Healthcare; and being a member of advisory boards for Astellas, Astrazeneca, and Vifor Pharma. J-JC reports funding to Karolinska Institutet by AstraZeneca, Astellas, Amgen, Vifor Pharma, and NovoNordisk; personal honoraria for lectures by Fresenius Kabi, Baxter Healthcare, and Abbott; and being a member of advisory boards for Astellas, AstraZeneca, and GSK. MS reports funding or institutional payment by the Swedish Kidney Foundation, VINNOVA (Swedish Government), Skane University Hospital Foundation, and Inga Britt and Arne Lundbergs stiftelse; consulting fees by Hansa Biopharma and Vfor; and personal honoraria for lectures by Otsuka. All the other authors declared no competing interests.

## Supplementary Material

Supplementary File (PDF) **Figure S1.** Flowchart. **Figure S2.** Study design. **Table S1.** CKD etiologies included or excluded from the Other-CKD. **Table S2.** Definitions of comorbidities, medications, and outcomes. **Table S3.** Crude absolute risks of adverse events associated with SLE/LN-CKD, primary glomerular diseases, and Other-CKD. **Table S4.** Incidence rates and hazard ratios (95% CI) of primary outcomes associated with SLE/LN-CKD versus primary glomerular diseases and Other-CKD. **Table S5.** Incidence rates and hazard ratios (95% CI) of secondary outcomes associated with SLE/LN-CKD versus primary glomerular diseases and Other-CKD. **Table S6.** Sensitivity analyses: hazard ratios (95% CI) of long-term outcomes associated with SLE/LN-CKD versus primary glomerular diseases and Other-CKD. **Table S7.** Steroids and immunosuppressive treatment in Other-CKD. **Table S8.** Subgroup analyses for the risk of KRT, stratified by sex. **STROBE Checklist.**

## Footnotes

Supplementary File (PDF)
**Figure S1.** Flowchart. **Figure S2.** Study design. **Table S1.** CKD etiologies included or excluded from the Other-CKD. **Table S2.** Definitions of comorbidities, medications, and outcomes. **Table S3.** Crude absolute risks of adverse events associated with SLE/LN-CKD, primary glomerular diseases, and Other-CKD. **Table S4.** Incidence rates and hazard ratios (95% CI) of primary outcomes associated with SLE/LN-CKD versus primary glomerular diseases and Other-CKD. **Table S5.** Incidence rates and hazard ratios (95% CI) of secondary outcomes associated with SLE/LN-CKD versus primary glomerular diseases and Other-CKD. **Table S6.** Sensitivity analyses: hazard ratios (95% CI) of long-term outcomes associated with SLE/LN-CKD versus primary glomerular diseases and Other-CKD. **Table S7.** Steroids and immunosuppressive treatment in Other-CKD. **Table S8.** Subgroup analyses for the risk of KRT, stratified by sex.
**STROBE Checklist**
