# Supplementary material for: Long-term Outcomes of Lupus Nephritis in Comparison to Other CKD Etiologies
Source: Kidney Int Rep. 2024 Oct 28;10(1):157–68. doi: 10.1016/j.ekir.2024.10.021 (PMC11725829; doi:10.1016/j.ekir.2024.10.021)
Supplement: Supplementary File (PDF) — Figure S1. Flowchart. Figure S2. Study design. Table S1. CKD etiologies included or excluded from the Other-CKD. Table S2. Definitions of comorbidities, medications, and outcomes. Table S3. Crude absolute risks of adverse events associated with SLE/LN-CKD, primary glomerular diseases, and Other-CKD. Table S4. Incidence rates and hazard ratios (95% CI) of primary outcomes associated with SLE/LN-CKD versus primary glomerular diseases and Other-CKD. Table S5. Incidence rates and hazard ratios (95% CI) of secondary outcomes associated with SLE/LN-CKD versus primary glomerular diseases and Other-CKD. Table S6. Sensitivity analyses: hazard ratios (95% CI) of long-term outcomes associated with SLE/LN-CKD versus primary glomerular diseases and Other-CKD. Table S7. Steroids and immunosuppressive treatment in Other-CKD. Table S8. Subgroup analyses for the risk of KRT, stratified by sex. STROBE Checklist. [file mmc1.pdf]

SUPPLEMENTARY MATERIAL

Long-term Outcomes of Lupus Nephritis in Comparison to Other CKD Etiologies

Charikleia Chrysostomou, Francesca Faustini, Iva Gunnarsson, Mårten Segelmark, Juan-Jesús Carrero, Peter Barany, Anne-Laure Faucon*, Marie Evans*

Table S1. CKD etiologies Included/ excluded from the Other-CKD group

**CKD etiologies included in the Other-CKD group**	**N = 34778**
Diabetic kidney disease	8877 (25.6%)
Hypertensive/Atherosclerotic	15088 (43.4%)
Chronic kidney disease of unknown etiology	7061 (20.3%)
Miscellaneous etiologies	3752 (10.7%)
**CKD etiologies excluded from the Other-CKD group**	**N = 6960**
Autoimmune / inflammatory CKD	2759 (39.6%)
Solid or hematologic malignancies	987 (14.1%)
Genetic diseases	2720 (39%)
Infectious diseases	335 (4.8%)
Other glomerulopathies	159 (2.2%)

Table S2. Definitions of comorbidities, medications, and outcomes

	ICD-10 codes	ATC codes
**Comorbidities**		
Diabetes mellitus	E10-E14	A10
Hypertension	I10-I15	
Dyslipidemia	E78	
Myocardial infarction	I21, I22, I25.2	
Coronary artery disease	I20-I25	
Heart failure	I11.0, I13.0, I13.2, I50	
Cerebrovascular disease	G45.9, I63, I64, I69	
Peripheral vascular disease	I70, I72, I73	
Arrythmia-Atrial Fibrillation	I47-I49	
Acute kidney injury	N17	
Depression-Anxiety	F32, F33, F41, F43	
Other coagulation disorders, including APS	D68.8, D68.6A	
Osteoporosis	M80, M81, M82.1, M82.8	
**Healthcare Utilization (12 months before inclusion)**		
AKI-diagnosis in hospitalization medical records	N17	
**Medications (6 months before inclusion)**		
Renin-angiotensin system inhibitors (ACEi, ARBs)		C09A, C09C
Calcium-channel blockers		C08C, C08D
Bêta-blockers		C07
Diuretics		C03A, C03B, C03C, C03D
Corticosteroids		H02
Mycophenolate Mofetil		L04AA06
Azathioprine		L04AX01
Calcineurine-Inhibitors		L04AD
Antiplatelet therapy		B01AC
VKA / Heparin		B01AA, B01AB
DOAKs		B01AE, B01AF
Lipid lowering drug		C10
**Outcomes**	**ICD-10 codes**	
All-cause mortality	Death in the Swedish “Cause of Death” Registry	
Initiation of kidney replacement therapy	Registration of the date of kidney transplantation or initiation of maintenance dialysis in the Swedish Renal Registry	
MACE	Death from cardiovascular cause, hospitalization for myocardial infarction, or stroke	
Death from cardiovascular cause	“I” family of the ICD-10 code, 4 first positions in the “Cause of death” Registry	
Hospitalization for myocardial infarction	I21, I22, I23, any position in the hospitalization medical records	
Hospitalization for stroke	I63, any position in the hospitalization medical records	
Acute kidney injury	N17, any position in the hospitalization medical records	
Heart Failure	I11.0, I13.0, I13.2, I50, any position in the hospitalization medical records	

ACEi: Angiotensin-converting enzyme inhibitors; ARBs: Angiotensin receptor blockers; APS: Antiphospholipid-Syndrome; ATC: Anatomic Therapeutic Chemical Classification System; CVD: cardiovascular disease; DOAKs: direct-anticoagulants; ICD-10: International Classification of Diseases tenth revision; MACE: major adverse cardiovascular event; VKA: Vitamin K antagonists.

Table S3. Crude absolute risks of adverse events associated with SLE/LN-CKD, primary glomerular diseases, and Other-CKD

	KRT, % (95%CI)	MACE, % (95%CI)	All-cause death, % (95%CI)
**1-year absolute risk**			
SLE/LN-CKD	6.59 (5.21 ; 7.97)	6.65 (5.34 ; 7.97)	5.32 (4.35 ; 6.28)
Primary glomerular diseases	10.73 (9.99 ; 11.47)	3.62 (3.25 ; 3.99)	2.93 (2.65 ; 3.21)
Other-CKD	7.26 (6.99 ; 7.53)	13.77 (13.41 ; 14.14)	11.37 (11.03 ; 11.71)
**5-year absolute risk**			
SLE/LN-CKD	23.23 (18.86 ; 27.6)	24.72 (20.37 ; 29.07)	27.06 (22.81 ; 31.3)
Primary glomerular diseases	37.02 (35.07 ; 38.97)	14.42 (13.07 ; 15.76)	15.77 (14.45 ; 17.1)
Other-CKD	22.57 (22.09 ; 23.05)	44.23 (43.65 ; 44.81)	50.19 (49.59 ; 50.78)
**10-year absolute risk**			
SLE/LN-CKD	31.58 (25.96 ; 37.19)	37.08 (31.14 ; 43.03)	47.01 (40.82 ; 53.21)
Primary glomerular diseases	50.04 (47.72 ; 52.37)	23.09 (21.06 ; 25.12)	29.22 (26.98 ; 31.46)
Other-CKD	27.99 (27.42 ; 28.57)	58.9 (58.22 ; 59.58)	75.41 (74.74 ; 76.08)

CKD: chronic kidney disease; KRT: kidney replacement therapy; MACE: major adverse cardiovascular events.

Table S4. Incidence rates and hazard ratios (95%CI) of primary outcomes associated with SLE/LN-CKD *versus* primary glomerular diseases and Other-CKD

	No. of events	Person-Years	IR per 100-PY (95% CI)	Unadjusted HR (95% CI)	Adjusted HR* (95% CI)
SLE/LN-CKD vs primary glomerular disease					
**All-cause mortality**					
Primary glomerular diseases	454	13473	3.4 (3.1-3.7)	1	1
SLE/LN-CKD	109	1763	6.2 (5.1-7.5)	1.84 (1.49-2.27)	1.64 (1.32-2.02)
**MACE**					
Primary glomerular diseases	376	12863	2.9 (2.6-3.2)	1	1
SLE/LN-CKD	89	1622	5.5 (4.4-6.8)	1.87 (1.49-2.36)	1.64 (1.3-2.07)
**KRT**					
Primary glomerular diseases	859	9525	9.0 (8.4-9.6)	1	1
SLE/LN-CKD	78	1450	5.4 (4.3-6.7)	0.61 (0.48-0.77)	0.81 (0.64-1.02)
SLE/LN-CKD vs Other-CKD					
**All-cause mortality**					
Other-CKD	18466	135200	13.7 (13.5-13.9)	1	1
SLE/LN-CKD	109	1763	6.2 (5.1-7.5)	0.45 (0.37-0.55)	0.98 (0.81-1.18)
**MACE**					
Other-CKD	15303	120204	12.7 (12.5-12.9)	1	1
SLE/LN-CKD	89	1622	5.5 (4.4-6.8)	0.46 (0.37- 0.57)	0.93 (0.76-1.15)
**KRT**					
Other-CKD	7453	111129	6.7 (6.6-6.9)	1	1
SLE/LN-CKD	78	1450	5.4 (4.3-6.7)	0.88 (0.70-1.10)	0.96 (0.76-1.21)

*Cox models were adjusted for age, sex, hypertension, diabetes, dyslipidemia, history of cardiovascular disease (ischemic heart disease, cerebrovascular disease, peripheral artery disease, heart failure, arrhythmia), acute kidney injury, systolic and diastolic blood pressure, body mass index, serum albumin, eGFR, renin-angiotensin system inhibitors, aspirin, vitamin K antagonists /Heparin, direct oral anticoagulants, healthcare utilization within 12 months prior to index date (all-cause hospitalizations, number of hospitalizations) and calendar year.

CI: confidence interval; CKD: chronic kidney disease; HR: hazard ratio; IR: incidence rate; KRT: kidney replacement therapy; MACE: major adverse cardiovascular events; PY: person-year; SLE/LN: systemic lupus erythematosus / lupus nephritis.

Table S5. Incidence rates and hazard ratios (95%CI) of secondary outcomes associated with SLE/LN-CKD *versus* primary glomerular diseases and Other-CKD.

	No. of events	Person-Years	IR per 100-PY (95% CI)	Unadjusted HR (95% CI)	Adjusted HR* (95% CI)
SLE/LN-CKD s vs primary glomerular disease					
**Cardiovascular mortality**					
Primary glomerular diseases	242	13473	1.8 (1.6-2.0)	1	1
SLE/LN-CKD	60	1763	3.4 (2.6-4.4)	1.90 (1.43-2.52)	1.67 (1.26-2.22)
**Non-CV mortality**					
Primary glomerular diseases	212	13473	1.6 (1.4-1.8)	1	1
SLE/LN-CKD	49	1763	2.8 (2.1-3.7)	1.76 (1.29-2.41)	1.62 (1.18-2.21)
**Myocardial infarction**					
Primary glomerular diseases	101	13163	0.8 (0.6-0.9)	1	1
SLE/LN-CKD	27	1682	1.6 (1.1-2.3)	2.09 (1.37-3.20)	2.07 (1.35-3.17)
**Stroke**					
Primary glomerular diseases	133	13117	1.0 (0.8-1.2)	1	1
SLE/LN-CKD	30	1699	1.8 (1.2-2.5)	1.74 (1.17-2.58)	1.56 (1.04-2.32)
**Heart failure**					
Primary glomerular diseases	304	12796	2.4 (2.1-2.7)	1	1
SLE/LN-CKD	83	1606	5.2 (4.1-6.4)	2.15 (1.69-2.74)	1.67 (1.31-2.13)
**AKI**					
Primary glomerular diseases	189	12945	1.5 (1.3-1.7)	1	1
SLE/LN-CKD	39	1705	2.3 (1.6-3.1)	1.56 (1.11-2.20)	1.46 (1.03-2.07)
SLE/LN-CKD vs Other-CKD					
**CV mortality**					
Other-CKD	12034	135200	8.9 (8.7-9.1)	1	1
SLE/LN-CKD	60	1763	3.4 (2.6-4.4)	0.39 (0.30-0.50)	0.90 (0.70-1.16)
**Non-CV mortality**					
Other-CKD	6432	135200	4.8 (4.6-4.9)	1	1
SLE/LN-CKD	49	1763	2.8 (2.1-3.7)	0.56 (0.42-0.74)	1.10 (0.83-1.47)
**Myocardial infarction**					
Other-CKD	3720	128493	2.9 (2.8-3.0)	1	1
SLE/LN-CKD	27	1682	1.6 (1.1-2.3)	0.60 (0.41-0.87)	1.28 (0.87-1.88)
**Stroke**					
Other-CKD	4573	125792	3.6 (3.5-3.7)	1	1
SLE/LN-CKD	30	1699	1.8 (1.2-2.5)	0.54 (0.38-0.77)	0.93 (0.64-1.33)
**Heart failure**					
Other-CKD	12985	112754	11.5 (11.3-11.7)	1	1
SLE/LN-CKD	83	1606	5.2 (4.1-6.4)	0.53 (0.43-0.66)	1.06 (0.85-1.31)
**AKI**					
Other-CKD	3921	129130	3.0 (2.9-3.1)	1	1
SLE/LN-CKD	39	1705	2.3 (1.6-3.1)	0.80 (0.58-1.10)	1.08 (0.78-1.48)

* Cox models were adjusted for age, sex, hypertension, diabetes, dyslipidemia, history of cardiovascular disease (ischemic heart disease, cerebrovascular disease, peripheral artery disease, heart failure, arrhythmia), acute kidney injury, systolic and diastolic blood pressure, body mass index, serum albumin, eGFR, renin-angiotensin system inhibitors, aspirin, vitamin K antagonists/Heparin, direct oral anticoagulants, healthcare utilization within 12 months prior to index date (all-cause hospitalizations, number of hospitalizations) and calendar year.

CI: confidence interval; CKD: chronic kidney disease; HR: hazard ratio; IR: incidence rate; KRT: kidney replacement therapy; MACE: major adverse cardiovascular events; PY: person-year; SLE/LN: systemic lupus erythematosus / lupus nephritis.

Table S6. Sensitivity analyses: hazard ratios (95%CI) of long-term outcomes associated with SLE/LN-CKD *versus* primary glomerular diseases and Other-CKD

	Adjusted HR (95% CI)<br><br>All SLE/LN-CKD<br><br>- Main analysis -	Adjusted HR (95% CI)<br>+ albuminuria<br>All SLE/LN-CKD<br><br>- Sensitivity analysis -	Adjusted HR (95% CI)<br><br>LN-CKD identified with the ERA coding system<br>- Sensitivity analysis -
**SLE/LN-CKD *vs.* primary glomerular diseases**			
All-cause mortality	1.63 (1.32-2.02)	1.69 (1.37-2.09)	1.44 (1.11-1.88)
MACE	1.65 (1.31-2.08)	1.69 (1.34-2.14)	1.66 (1.26-2.20)
KRT	0.81 (0.64-1.02)	0.81 (0.64-1.04)	0.73 (0.56-0.95)
**SLE/LN-CKD *vs*. Other-CKD**			
All-cause mortality	0.98 (0.81-1.18)	0.96 (0.79-1.16)	0.86 (0.67-1.10)
MACE	0.94 (0.76-1.16)	0.91 (0.74-1.13)	0.95 (0.73-1.23)
KRT	0.96 (0.76-1.21)	0.80 (0.63-1.02)	0.86 (0.66-1.12)

Cox models were adjusted for age, sex, hypertension, diabetes, dyslipidemia, history of cardiovascular disease (ischemic heart disease, cerebrovascular disease, peripheral artery disease, heart failure, arrhythmia), acute kidney injury, systolic and diastolic blood pressure, body mass index, serum albumin, eGFR, renin-angiotensin system inhibitors, aspirin, vitamin K antagonists /Heparin, direct oral anticoagulants, healthcare utilization within 12 months prior to index date (all-cause hospitalizations, number of hospitalizations) and calendar year.

CI: confidence intervals; CKD: chronic kidney disease; ERA: European Renal Association; HR: hazard ratio; KRT: kidney replacement therapy; MACE; major adverse cardiovascular events; LN: lupus nephritis; SLE/LN: systemic lupus erythematosus / lupus nephritis.

Table S7: Steroids and immunosuppressive treatment in patients with Other-CKD

Table A: Corticosteroid users in Other-CKD

	**Corticosteroid users<br>13.7% of Other-CKD**
Addison disease	49
Chronic pulmonary disease*	1289
Inflammatory bowel / liver disease	240
Gout	2005
Hematologic malignancy	166
Non-kidney organ ttransplantation	309
Tubulointerstitial nephropathy	322

*Includes: asthma, COPD, emphysema, bronchiectasis, interstitial lung disease.

Table B: Immunosuppressive therapy – users in Other-CKD

	**Immunosuppressive therapy – users<br>2.1% of Other-CKD**
Non-kidney organ transplantation	374
Chronic pulmonary disease*	186
Bowel/liver disease	87
Articular disease	175

*Includes: asthma, COPD, emphysema, bronchiectasis, interstitial lung disease.

Table S8. Subgroup analysis for the risk of KRT, stratified on sex

	Crude HR 95%CI			Adjusted HR 95%CI		
**KRT**	**Overall**	**Women**	**Men**	**Overall**	**Women**	**Men**
**LN vs PGD**	0.61 (0.48-0.77)	0.84 (0.64 - 1.12)	0.46 (0.28 - 0.76)	0.81 (0.64-1.02)	0.97 (0.73 - 1.30)	0.52 (0.32 - 0.86)
**LN vs Other-CKD**	0.88 (0.70- 1.10)	1.08 (1.04 - 1.12)	0.68 (0.64 - 0.73)	0.96 (0.76-1.21)	1.02 (0.76 - 1.36)	0.50 (0.30 - 0.82)

CKD: chronic kidney disease; HR: hazard ratio; KRT: kidney replacement therapy; LN: lupus nephritis; PGD: primary glomerular diseases; SLE/LN: systemic lupus erythematosus / lupus nephritis

Figure S1. Flowchart

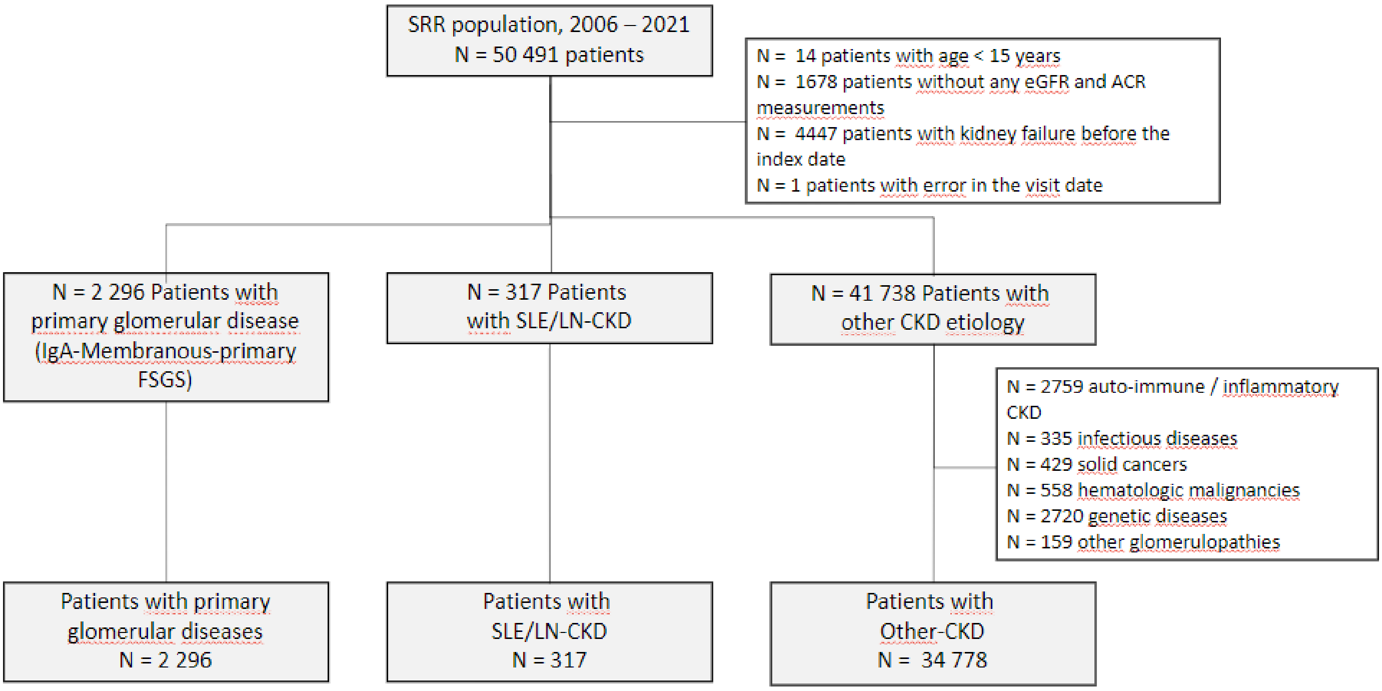

Figure S2. Study Design

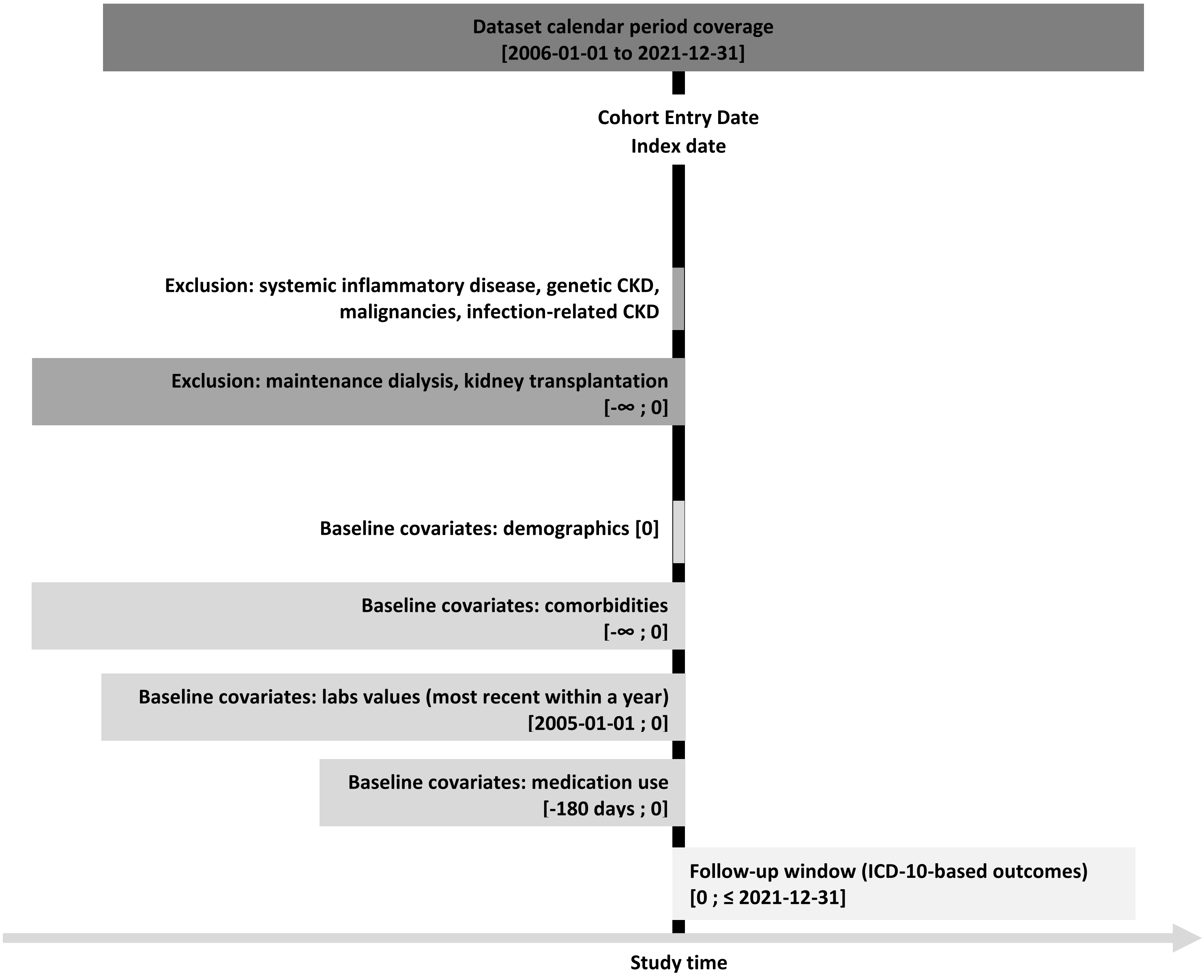

This graphical depiction of study design shows the different time windows used to define exposure SLE/LN-CKD vs Primary Glomerular Diseases and vs Other-CKD, exclusion criteria, baseline covariates and outcomes. Adapted from *Schneeweiss et al, Ann Int Med, 2019*.

STROBE Statement – The Strengthening the Reporting of Observational Studies in Epidemiology

	Item No	Recommendation	Page
**Title and abstract**	1	(*a*) Indicate the study's design with a commonly used term in the title or the abstract	1
		(*b*) Provide in the abstract an informative and balanced summary of what was done and what was found	3
**Introduction**			
Background/rationale	2	Explain the scientific background and rationale for the investigation being reported	5
Objectives	3	State specific objectives, including any prespecified hypotheses	5
**Methods**			
Study design	4	Present key elements of study design early in the paper	6
Setting	5	Describe the setting, locations, and relevant dates, including periods of recruitment, exposure, follow-up, and data collection	6
Participants	6	(*a*) *Cohort study*—Give the eligibility criteria, and the sources and methods of selection of participants. Describe methods of follow-up<br>*Case-control study*—Give the eligibility criteria, and the sources and methods of case ascertainment and control selection. Give the rationale for the choice of cases and controls<br>*Cross-sectional study*—Give the eligibility criteria, and the sources and methods of selection of participants	6
Variables	7	Clearly define all outcomes, exposures, predictors, potential confounders, and effect modifiers. Give diagnostic criteria, if applicable	7
Data sources/ measurement	8	For each variable of interest, give sources of data and details of methods of assessment (measurement).	6,7
Bias	9	Describe any efforts to address potential sources of bias	6-8
Study size	10	Explain how the study size was arrived at (if applicable)	NA
Quantitative variables	11	Explain how quantitative variables were handled in the analyses. If applicable, describe which groupings were chosen and why	NA
Statistical methods	12	(*a*) Describe all statistical methods, including those used to control for confounding	7,8
		(*b*) Describe any methods used to examine subgroups and interactions	8
		(*c*) Explain how missing data were addressed	8
		(*d*) *Cohort study*—If applicable, explain how loss to follow-up was addressed<br>*Case-control study*—If applicable, explain how matching of cases and controls was addressed<br>*Cross-sectional study*—If applicable, describe analytical methods taking account of sampling strategy	NA
		(*e*) Describe any sensitivity analyses	8
**Results**			
Participants	13	(a) Report numbers of individuals at each stage of study—eg numbers potentially eligible, examined for eligibility, confirmed eligible, included in the study, completing follow-up, and analyzed	9
		(c) Use of a flow diagram	Fig.S1
Descriptive data	14	(a) Give characteristics of study participants (eg demographic, clinical, social) and information on exposures and potential confounders	Page 9<br>Tab.1
		(b) Indicate number of participants with missing data for each variable of interest	Tab.1
		(c) *Cohort study*—Summarise follow-up time (eg, average and total amount)	9
Outcome data	15	*Cohort study*—Report numbers of outcome events or summary measures over time	Page 9<br>Tab.S4
		Case-control study—Report numbers in each exposure category, or summary measures of exposure	NA
		Cross-sectional study—Report numbers of outcome events or summary measures	NA
Main results	16	(*a*) Give unadjusted estimates and, if applicable, confounder-adjusted estimates and their precision (eg, 95% confidence interval). Make clear which confounders were adjusted for and why they were included	9,10<br>Tab S4,<br>S5<br>Fig 1-3

Other analyses	17	Report other analyses done—eg analyses of subgroups and interactions, and sensitivity analyses	7,8,9 Tab S6, S8
**Discussion**			
Key results	18	Summarise key results with reference to study objectives	11
Limitations	19	Discuss limitations of the study, taking into account sources of potential bias or imprecision. Discuss both direction and magnitude of any potential bias	12
Interpretation	20	Give a cautious overall interpretation of results considering objectives, limitations, multiplicity of analyses, results from similar studies, and other relevant evidence	11-13
Generalisability	21	Discuss the generalisability (external validity) of the study results	11-13
